# Systemic Responses Towards Oxy-Inflammation, Hormones, and Mood in Breast Cancer Survivors: Preliminary Evidences from Dragon Boat Endurance Race

**DOI:** 10.3390/jcm14072532

**Published:** 2025-04-07

**Authors:** Michela Montorsi, Alessandra Vezzoli, Federica Mrakic Sposta, Maristella Gussoni, Andrea Brizzolari, Gerardo Bosco, Cinzia Dellanoce, Alessandra Barassi, Barbara Picconi, Cristina Ranuncoli, Simona Mrakic Sposta

**Affiliations:** 1 Department of Human Sciences and Promotion of the Quality of Life, San Raffaele Roma Open University, 00166 Roma, Italy; michela.montorsi@uniroma5.it (M.M.); barbara.picconi@uniroma5.it (B.P.); 2 Institute of Clinical Physiology, National Research Council (IFC-CNR), 20162 Milan, Italy; maristella.gussoni@unimi.it (M.G.); cinziacarla.dellanoce@cnr.it (C.D.); 3 Department of Biomedical Sciences, University of Padova, 35131 Padova, Italy; andreabrizzolari79@gmail.com (A.B.); gerardo.bosco@unipd.it (G.B.); 4 IRCCS Humanitas Research Hospital, Via Manzoni 56, Rozzano, 20089 Milan, Italy; federica.mrakic_sposta@humanitas.it; 5 Department of Health Sciences, Università degli Studi of Milan, 20100 Milan, Italy; alessandra.barassi@unimi.it; 6 Scuola Regionale dello Sport CONI Valle d’Aosta, 11100 Aosta, Italy; cristina.ranuncoli@scuola.istruzione.it

**Keywords:** oxidative stress, inflammation, hormones, women, breast cancer, dragon boat, exercise, endurance, mood, quality of life

## Abstract

**Background/Objectives:** Endurance exercise increases oxygen demand and, when not balanced by antioxidant defenses, consequently, oxidative stress and inflammatory cytokines increase too. In breast cancer survivors (BCS), post-treatment, physical capacity decreases, lowering life quality. Dragon boat (DB) paddling has shown benefits in reducing lymphedema and improving psychological well-being. This study aimed at non-invasively investigating in BCS, by means of saliva and urine samples, the systemic responses to oxy-inflammation, and appetite hormones after a DB endurance race. **Methods**: 15 BCS and 15 healthy women (5 (CTR) who performed the DB race too) were studied. BCS and CTR were monitored pre- and post-race. Reactive oxygen species (ROS) production, total antioxidant capacity (TAC), lipid peroxidation (8-iso), DNA oxidation (8-OH-dG), nitric oxide metabolites (NOx), inflammation markers (IL-6-10 and TNF*α*), appetite hormones, electrolytes concentration, psychometric, and physical scales were assessed. **Results**: At rest, compared to healthy women, BCS showed a significant increase in oxy-inflammation biomarkers. BCS showed a general increase in oxy-inflammation parameters compared to CTR after the DB race. In BCS, there were the following results: ROS: +80%; lipid peroxidation: +103%; DNA oxidation: +44%; interleukins-6: +179%; IL-10: +55%; TNFα: +9%, NOx: +60% increases and unbalanced appetite hormones: leptin (−32%); and ghrelin (+53%). Moreover, the dragon boat offered a holistic approach to recovery, addressing emotional and social needs supporting belonging, love, and esteem needs, reported to be about 56% of the motivations in this activity, while post-race the following increased: a sense of fatigue (+55%); tiredness (48%); a cold sensation (+15%); and +32% pain. **Conclusions**: This study provided evidence that, in BCS, a DB endurance race produces an important imbalance in the oxy-inflammation state, at the same time being accompanied by a positive impact on subjective mood and general wellness. Future studies should focus on long-term effects.

## 1. Introduction

Endurance exercise, characterized by continuous and prolonged physical activity, enhances the demand for oxygen in muscles, thus increasing reactive oxygen species (ROS) levels [1]. While moderate exercise stimulates antioxidant defense mechanisms, excessive or intense exercise without adequate recovery can exacerbate oxidative stress (OxS) and cause damage to muscle tissue [2]. OxS arises when an imbalance between ROS production and the body’s ability to neutralize them using antioxidants takes place. This process can lead to cellular damage, affecting lipids, proteins, and DNA, and has been implicated in various diseases, including cancer, cardiovascular orders, and metabolic conditions [3]. Otherwise, in the context of physical activity, training increases the antioxidant defense system accompanied by a decrease in the exercise-induced oxidative stress [4,5,6]. As a consequence of physical exercise, some cytokines are produced by muscle contraction, for example, IL-6 and IL-10 increase [5]. Moreover, a linear increase of IL-10 has been seen in relation to a rise in exercise duration [7,8]. Also, appetite regulation that involves a sophisticated interplay of hormones is affected by exercise and energy balance [9,10].

Breast cancer is an important global health problem. In the latest report of the World Health Organization (WHO) [11], in 2022, the incidence in Southern Europe was 81.8% with 13.8% mortality. After diagnosis and during treatments there is a reduction in individual physical capacity with a consequent reduction in the quality of life (QoL). Most of these cancers (70–80%) express an estrogenic receptor, so a five-year hormone therapy to reduce the estrogenic level is indicated. This treatment results in further impairment of musculoskeletal activity [12]. One of the post-operative complications of breast cancer is lymphedema, due to the removal of the axillary lymph nodes on the operated side, which leads to the retention of lymphatic fluid in the interstitial tissues. Regular physical exercise helps to prevent lymphedema. In particular, it has been seen that the activity carried out on the dragon boat (DB) is specific for women operated on for breast cancer [13]. It has now been demonstrated how important physical activity is for improving one’s quality of life thanks to the positive effects not only on the body, but also on cognitive functions [14,15]. In these contexts, the discipline of DB is inserted.

Dragon boat discipline was born in China more than 2000 years ago, but it was only in 1998 that McKenzie [16] published the first study on breast cancer survivors (BCS) who participated in the Vancouver Dragon Boat Festival. Two years before, he created the first DB team constituted by BCS to prove the positive effect of this kind of sport on lymphedema and, in general, on the well-being of women with breast cancer.

A dragon boat is a long canoe with 20 seats (10 on each side) with a drummer at the bow and a helmsman at the stern. In addition to the positive effects on the upper limbs, for those with lymphedema, paddling on a DB plays also beneficial effects on a psychological level and in terms of social relationships among women sharing the same problems and helping each other [13]. A study on elite rowers, a sport comparable to dragon boating, demonstrated increased markers of OxS following high-intensity exercise [6]. Many studies report the importance of physical activity in both physiological and pathological conditions (i.e., cancer patients) [17], but few studies have been carried out regarding the changes in oxidative stress and inflammatory status in patients with breast cancer who practice the dragon boat sport [18].

Considering the importance of exercise, social relationships, the psychological health benefit, and the lack of data in the scientific literature, women who are breast cancer survivors’ (BCS) pre- and post-extreme physical activity, that is an endurance race, was studied. The aim of this observational study was to monitor a breast cancer survivors (BCS) group through non-invasive methods (saliva and urine samples), oxidative stress, inflammation, and hormones of appetite changes before and after a dragon boat non-competitive endurance race (Vogalonga 2023—Venice). Subjective mood and general wellness were also evaluated to determine the effects of the dragon boat on the well-being of breast cancer athletes.

## 2. Materials and Methods

### 2.1. Study Design

**Figure 1:**
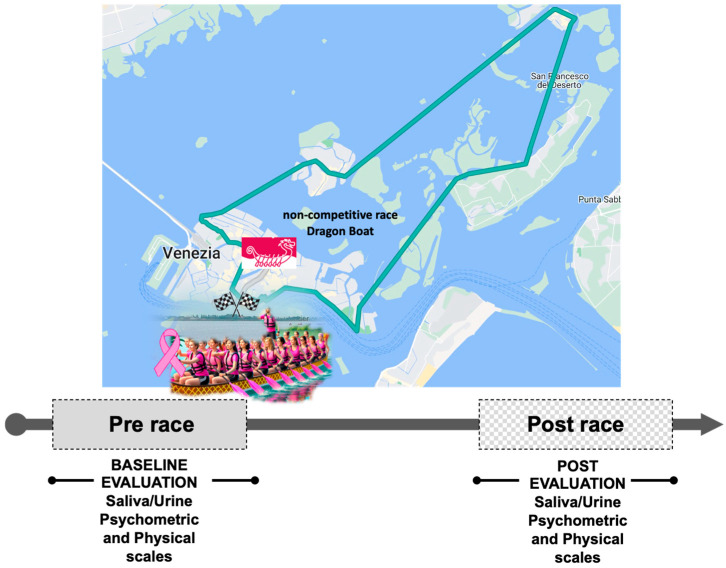
Sketch of the experimental protocol and map showing the navigation route. Data were collected before departure (Pre-Race), and Post Race “Vogalonga 2023”. Map was obtained by Google, Inst. Geogr. National. Race route Vogalonga 2023: bacino di San Marco, canale delle Navi, canale della Bissa, canale Passaora, canale Crevan, Burano, Mazzorbo, canale S. Giacomo, canale Scomenzera, canale Bisatto, Murano, canale di Tessera, rio di Cannaregio, Canal Grande, Rialto, Punta della Salute.

The study conducted on “Breast Cancer Survivors’ Dragon Boat Team” was performed in 2023 during the “47th Vogalonga”, a non-competitive paddling/rowing race of approximately 32 km through the city of Venice and the lagoon, up to Burano, and returning to Venice passing through Murano. In Figure 1, the route of the race and the sketch of the experimental protocol are reported.

The only rule of the race is the use of rowing boats, without any restrictions regarding weight, size, or the number of rowers. “Vogalonga” is the most important non-competitive regatta in Venice—open to all types of rowing boats—and is a tribute to the navigation techniques of the past (from 1974), an event created to promote the use of sustainable boats and to protect the city of Venice from wave motion. The crew object of this study performed the race in 5 h, with a cruising speed of about 6 km per hour and an average of 50 paddles per minute.

The weather was partly cloudy with an average temperature of 22 °C (min 19 °C–max 25 °C), humidity: 58%, average wind speed: 10 km/h, and sea level pressure: 1014 mb.

### 2.2. Participants

This study was conducted following the Declaration of Helsinki and was approved by the Bio-Ethical Committee of the University of Milan (Aut. n 37/17). Informed consent was obtained from all subjects involved in the study. The crew was made up of a helmsman and 20 women, 15 of them with a breast cancer history (BCS), whose characteristics are reported in Table 1. The remaining part of the crew was made up of 5 healthy women (CTR).

To compare the basal values of the selected biomarkers, a control group of heathy athletic women (HFS n = 15), age 50.67 ± 4.25 years; body weight 59.67 ± 7.65 kg; height 165.10 ± 1.98 cm; and BMI 21.87 ± 2.56 kg.m^−2^, was enrolled too. Five women from this group were the components of the crew examined and they were tested pre- and post-race, too.

All participants have obtained a certificate of medical sports suitability for competitive activity issued by the Italian Medical Sport Federation, FIMS/CONI. In the healthy group, the exclusion criteria comprised alcohol, obesity, smoking, current use of medicines, special diet, minerals, vitamins or other kind of supplementation, as well as antioxidant supply.

### 2.3. Sample Collection

Saliva and urine samples were collected from all participants just before (at 8 a.m.: pre-race) and, from all crew components, immediately after the race (about 3 p.m.; post-race). Saliva samples were obtained using Salivette collection devices (Sarstedt Nümbrecht); the samples were centrifuged for 5 min at 1000× *g* and stored at –80 °C for further analyses.

Urine samples were contemporarily collected in sterile containers by voluntary voiding and were stored at 4 °C in a portable cooler and during the transport back to the laboratory. The specimens were then stored in multiple aliquots at −80 °C until assayed and thawed only once before analysis.

### 2.4. Biomarkers

#### 2.4.1. ROS Detection by Electron Paramagnetic Resonance

An electron paramagnetic resonance spectrometer, operating in X-band (E-Scan, Bruker Co., Manning Park Billerica, MA, USA) at 9.3 GHz was used to measure and quantitate ROS production. Samples (saliva) were stabilized at 37 °C using a Temperature and Gas Controller “Bio III” unit (Noxigen Science Transfer & Diagnostics GmbH, Elzach, Germany), interfaced with the E-Scan. ROS detection methods were previously described [19,20]. Spin probe 1-hydroxy-3-methoxycarbonyl-2,2,5,5tetramethyl-pyrrolidine-hydrochloride (CMH, Noxygen Science Transfer & Diagnostics, Germany) (1 mM) was used. The EPR signal, intrinsically proportional to the number of unpaired electrons of the investigated specimen, can be converted into absolute concentration (μmol·min^−1^) by using the stable CP• (3-Carboxy-2,2,5,5-tetramethyl-1-pyrrolidinyloxy) compound as a reference. By using this approach, the absolute quantification of radical species is obtained. The high reproducibility of the method has been previously demonstrated [4].

#### 2.4.2. Total Antioxidant Capacity (TAC)

The Trolox-equivalent antioxidant capacity (TAC) assay is a commonly used commercial method (Cayman Chemical, Ann Arbor, Michigan, USA, Item No. 709001) and was employed to measure TAC levels. The procedure followed the manufacturer’s instructions and established protocols [21].

#### 2.4.3. Oxidative Damage in Urine

Lipid peroxidation was assessed in urine by measuring 8-isoprostane concentration (8-iso-PGF2α) (Cayman Chemical, Ann Arbor, Michigan, USA, Item No. 516351). The method was previously described [19,22]. DNA damage (urinary concentrations of 8-OH-dG) was measured using a commercial ELISA kit (Cayman Chemical, Ann Arbor, Michigan, USA, Item No. 89320).

The samples were read in duplicate at a wavelength 412 nm and concentration determined using standard curves as previously described. The coefficient of variation (CV) was indicated by the manufacturer: inter-assay CV 11.2% and 10.7% and intra-assay CV 14.6% and 11.6%, respectively, for 8-iso-PGF2α and 8-OH-dG.

#### 2.4.4. Quantification of Inflammatory Markers in Saliva

IL-6, IL-10, and TNF-a saliva levels were determined by ultrasensitive ELISA immunoassays (Fine Test, Wuhan, China, Cat No.: EH0201; EH0173; EH0302), according to the manufacturer’s instructions [20,22]. The assays are based on a double-antibody sandwich technique. Saliva levels of inflammatory markers in pg/mL were calculated. The samples’ concentrations were determined at 450 nm. The coefficient of variation (CV) was indicated by the manufacturer for IL-6, IL-10, and TNFα: inter-assay CV 4.62%, 5.26%, and 5.56% and intra-assay CV 5.35%, 5.05%, and 5.53%, respectively.

#### 2.4.5. No Metabolites (Nitrite and Nitrate) in Urine

NO_2_ + NO_3_ = NO metabolites levels were assessed in urine by using a method based on the Griess reaction, using a commercial kit (Cayman Chemical, Ann Arbor, Michigan, USA, Item No. 780001) [19]. The coefficient of variation (CV) was indicated by the manufacturer: inter-assay CV 3.4% and intra-assay CV 2.7%.

#### 2.4.6. Appetite Hormones

Salivary leptin, ghrelin, and Insulin-like Growth Factor-1 (IGF-1) levels were measured using enzyme-linked immunosorbent assay (ELISA kit cat. No.: EH0216, EH0355 and EH0165 FineTest, Wuhan, China) kits, following the manufacturer’s instructions [20,23,24]. The inter-assay coefficient of variation was in the range indicated by the manufacturer: leptin 5.73%, ghrelin 4.23%, and IGF-1 5.03%. Every assessment was carried out in duplicate and read at 450 nm by a microplate reader spectrophotometer (Infinite M200, Tecan Group Ltd., Männedorf, Switzerland).

#### 2.4.7. Creatinine, and Neopterin in Urine

Urinary creatinine and neopterin concentrations were measured in duplicate using isocratic high-pressure liquid chromatography. The calibration curves were linear for neopterin (0.125–1 μmol/L) and creatinine (1.25–10 mmol/L). The inter-assay and intra-assay variation coefficients were below 5%. The methods have been previously described [19,25].

#### 2.4.8. Uric Acid and Electrolytes

Urea, sodium (Na^+^), potassium (K^+^), chlorine (Cl^−^), magnesium (Mg^2+^), phosphorus (P), calcium (Ca^2+^), and uric acid were investigated in urine samples before and after the race. The method was previously described [26]. After thawing and vortexing the samples, 500 µL of each was placed in a plastic test tube. The test tubes were allocated in the autosampler of a Roche Cobas^®^ 6000 analyzer (Roche Diagnostics, Basel, Switzerland). The reported total imprecision was <2.8%, while the intra-assay CV% was <1.8%.

#### 2.4.9. Psychometric and Physical Scale

The quality of recovery was assessed before and after the race by using the Total Quality of Recovery scale (TQR) proposed by Kenttä and Hassmén [26,27].

Perceived exertion was evaluated immediately after the race based on physical sensations, with muscle fatigue assessed using the Borg Rate of Perceived Exertion scale. (RPE) [26].

To assess subjective mood, overall wellness (happy/unhappy, rested/tired), general sensations (hot/cold, calm/agitated), and head pain/no pain, a 0–100 mm visual analog scale (VAS) was used. If pain was reported, its location was also identified [26,28].

The Profile of Mood States (POMS) is a widely used instrument among sports psychologists, employed to assess and contrast the dominant emotional states of elite athletes and non-athletes. In short-form, this tool was administered to measure certain psychological traits, focusing on tension–anxiety, depression–dejection, anger–hostility, vigor–activity, fatigue–inertia, and confusion– bewildered on a five-point scale from 0 to 4 [26,29].

High vigor scores reflect a good mood or emotion, and low scores on the other subscales reflect a good mood or emotion.

Also, the Maslow’s pyramid of human motivation was assessed. The five levels in Maslow’s pyramid were investigated. From the bottom of the hierarchy upwards, the needs are: physiological (food and clothing), safety (job security), love and belonging needs (friendship), esteem, and self-actualization [30].

The psychological factors were determined by adopting the European Organization for Research and Treatment of Cancer (EORTC) Quality of Life Group Core-30 (QLQ-C30, version 3), which is one of the most widely used tools to measure the quality of life in oncologic patients. The method assesses various aspects, including physical, emotional, and social functions, as well as symptoms such as fatigue, pain, dyspnea, and insomnia [31,32].

Finally, the Life enhancement sub-scale was performed to test the perceived benefits of the dragon boat activity.

### 2.5. Statistical Analysis

Data are presented as mean ± SD. Statistical analyses were performed using non-parametric tests with the GraphPad Prism package (GraphPad Prism 10.3.1, GraphPad Software Inc., San Diego, CA, USA) for Mac. The normality of the data distribution was tested with the Shapiro–Wilk test. Data were compared by using ANOVA multiple comparison followed by the FDR method of Benjamini and Hochberg to further check the groups’ significance (* = q < Q) for ROS, TAC, TNF-α; 8-iso; 8-OH-dG, IL-6, IL-10, NOx, NO_2_, creatinine, neopterin, uric acid, leptin ghrelin, and uric acid. The confidence interval (CI) for the estimated values was calculated. dCohen with 95%CI was used for calculating the size effect. Taking the first measurement Pre (BCS or CTR) as 100%, biological changes were calculated at the end of the race (Post_ BCS or CTR), thereby obtaining an appreciation of the magnitude of change rather than the absolute values. The psychometric scale and electrolytes value were compared by using ANOVA multiple comparison followed by Bonferroni’s multiple comparison tests.

A multiple Spearman correlation analysis was also performed. A *p* < 0.05 was considered statistically significant. Change Δ% estimation ((pre-value/post-value) × 100) is also reported.

The ROS production rate was considered as the primary outcome (no other parameters were taken into account) and prospective calculations of power to determine sample size were made using the G power software (GPower 3.1) [33]. At 80% power, the sample size—calculated in preliminary studies [34,35,36]—was set at eleven/thirteen subjects.

## 3. Results

### 3.1. Basal Biomarkers Concentration

**Figure 2:**
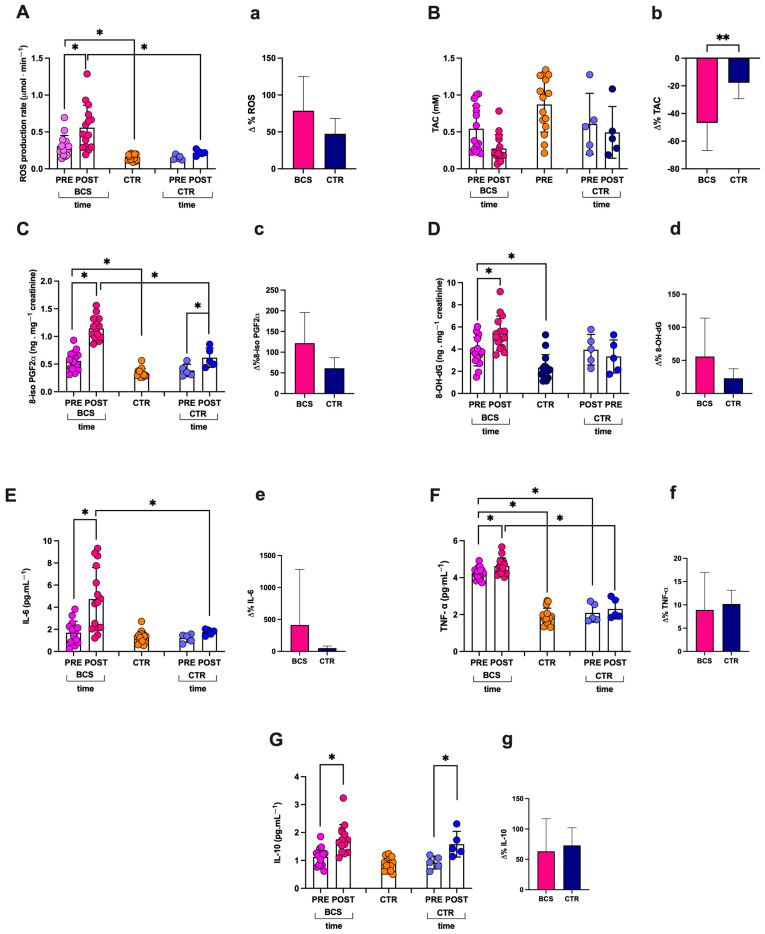
Histograms of: (**A** and **a**) ROS production and (**B** and **b**) TAC by saliva; (**C** and **c**) 8-iso PGF2α and (**D** and **d**) 8-OH-dG by urine; (**E** and **e**) IL-6, (**F** and **f**) TNF-α, and (**G** and **g**) IL-10 by saliva. In capital letters: values of samples concentrations collected pre- and post-race from the crew components (breast cancer survivors (BCS) and healthy participants (CTR)) and at rest from the HFS group. In lower letters: change Δ% estimation of BCS and CTR. Results are expressed as mean ± SD; * q < Q; ** *p* < 0.01.

**Figure 3:**
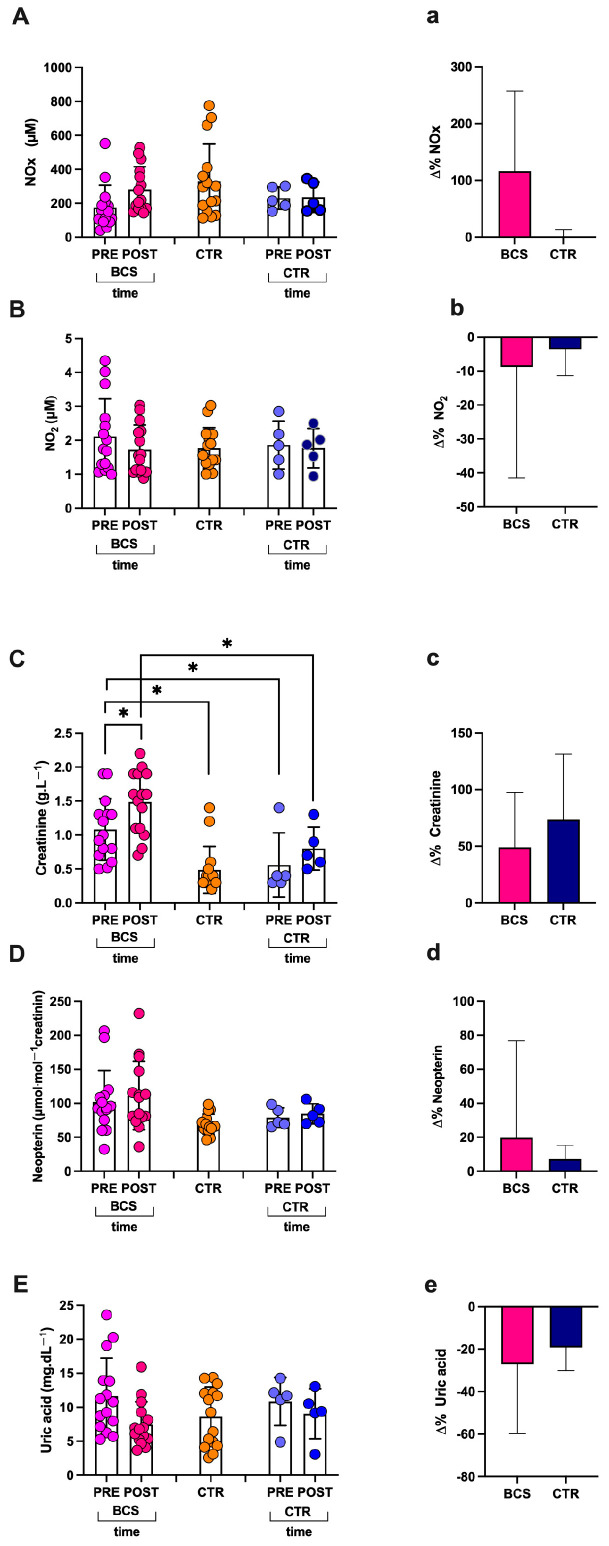
Histograms of (**A** and **a**) NO metabolites (NOx), (**B** and **b**) Nitrite (NO_2_), (**C** and **c**) creatinine, (**D** and **d**) neopterin, and (**E** and **e**) uric acid. In capital letters: values of sample concentrations pre- and post-race from the crew components (breast cancer survivors, BCS, and healthy CTR) and at rest for the HFS group. In lower letters: change Δ% estimation of BCS and CTR. Results are expressed as mean ± SD; * q < Q.

**Figure 4:**
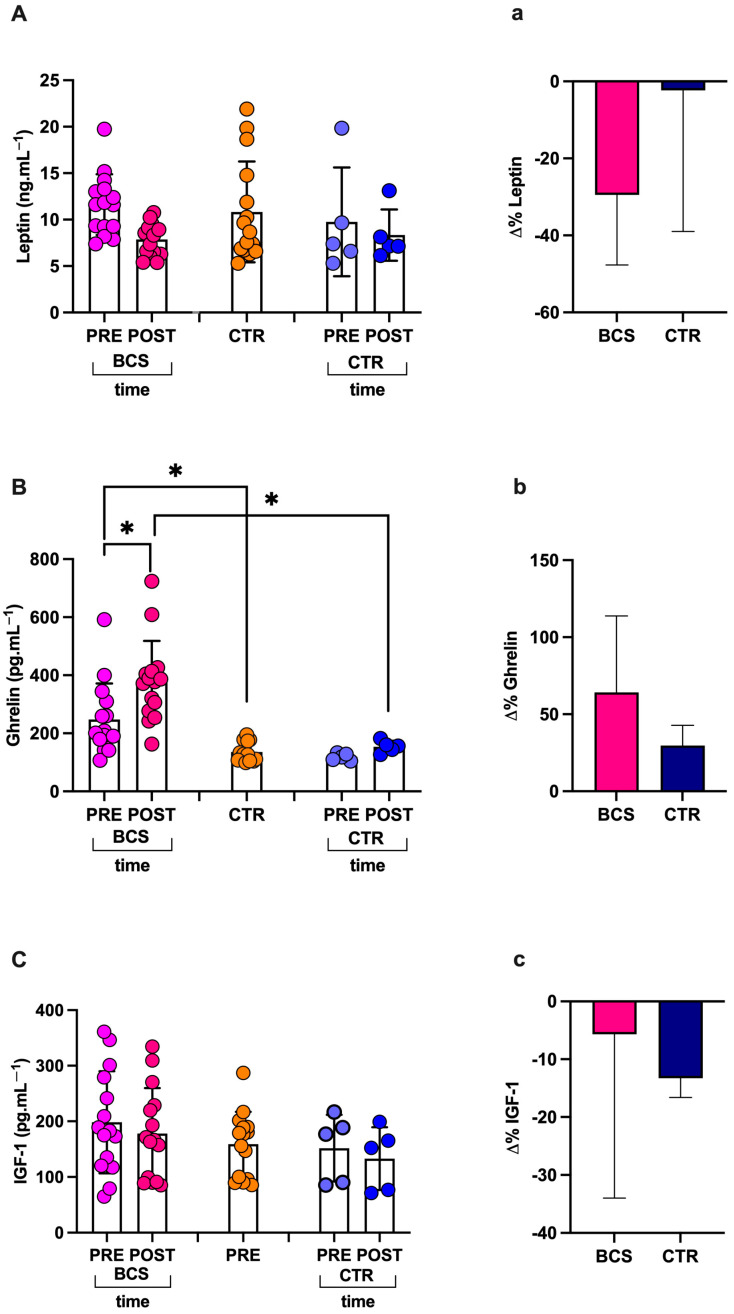
Histogram plots (mean ± SD) of (**A** and **a**) leptin, (**B** and **b**) ghrelin, and (**C** and **c**) IGF-1. In capital letters: values of samples concentrations collected pre- and post-race in crew components (breast cancer survivors, BCS, and healthy CTR) and at rest from the HFS group. In lower letters: change Δ% estimation of BCS and CTR. Significantly different results are expressed as mean ± SD; * q < Q.

In the present pilot, observational, non-invasive study, the oxy-inflammation levels of saliva and/or urine biomarkers were obtained pre- and post-race from the BCS group (n = 15). The number of subjects considered in this study was, of course, determined by the number of seats, 20, in the boat. Therefore, the control group that could be monitored, like the BCS women, pre- and post-race (CTR) was limited to the five aforementioned healthy women (see Section 2.2). With an awareness of the sample size being too little and not statistically significant, 10 healthy female athletes, comparable in age, BMI, and fitness level, were enrolled and added to CTR to form a control group at rest conditions of heathy female subjects (HFS n = 15). The results from all groups are shown in the Figures. Significant differences in biomarker concentrations between the BCS group collected pre-race and the HFS group were reported: in the BCS group, there was a higher level of ROS production (Figure 2A; +49%), lipids peroxidation (Figure 2C; +40%), DNA oxidation (Figure 2D; +42%), TNF-α (Figure 2F; +55%), creatinine (Figure 3C; +55%), and ghrelin (Figure 4B; +55%). No differences were found between the BCS group vs. the HFS group in TAC, IL-6, IL-10, NO_2_, Neopterin, Uric Acid, Leptin, and IGF-1.

### 3.2. Oxy-Inflammation and Total Antioxidant Post-Race Capacity

In the BCS group, significant increases post- vs. pre-race were calculated: (post vs. pre-race values; q-value; Lower and Upper 95% Coefficient Interval (CI); delta % respect to pre-race; and size effect dCohen) resulting, respectively, as follows: ROS production: 0.56 ± 0.30 vs. 0.31 ± 0.14 μmol·min^−1^; q = 0.0016; 0.389–0.727 and 0.226–0.387 95%CI; +80%; dCohen = 1.058 (Figure 2A); lipids peroxidation (8-iso PGF2α) 1.14 ± 0.20 vs. 0.56 ± 0.17 ng·mg^−1^ creatinine; q < 0.0001; 1.031–1.258 and 0.463–0.654 95%CI; +103%; dCohen = 3.105 (Figure 2C); DNA oxidation (8-OH-dG) 5.44 ± 1.54 vs. 3.77 ± 1.29 ng·mg^−1^ creatinine; q < 0.0075; 4.587–6.291 and 3.060–4.492 95%CI; and +44%; dCohen = 1.170 (Figure 2D). Also, biomarkers of inflammation increased post-race. IL-6: 4.74 ± 2.85 vs. 1.70 ± 1.03 pg·mL^−1^; q < 0.0001; 3.160–6.316 and 1.131–2.267 95%CI; +179%; dCohen = 1.419 (Figure 2E); TNF-α: 4.63 ± 0.45 vs. 4.25 ± 0.32 vs. pg·mL^−1^; q < 0.0303; 4.378–4.876 and 4.075–4.429 95%CI; +9%; dCohen = 0.968 (Figure 2F) and IL-10: 1.75 ± 0.53 vs. 1.13 ± 0.32 vs. pg·mL^−1^; q < 0.0002; 1.462–2.046 and 0.947–1.307 95%CI; +55%; and dCohen = 1.433 (Figure 2G). No significant differences in TAC were found: 0.27 ± 0.19 vs. 0.54 ± 0.31 mM; q > Q; 0.1692–0.3791 and 0.3692. 0.7162 95%CI; −50%; and dCohen = 1.0501 (Figure 2B).

In the CTR group (n = 5, the healthy women of the crew), significant differences in biomarker concentrations post- vs. pre-race were found in lipids peroxidation (8-iso PGF2α): 0.62 ± 0.18 vs. 0.38 ± 0.11 ng·mg^−1^ creatinine; q < 0.0001; 0.247–0.525 and 0.397–0.835 95%CI; +60%; dCohen = 1.606 (Figure 2C) and IL-10: 1.40 ± 2.31 vs. 0.61 ± 1.19 pg·mL^−1^; q < 0.0001; 0.637–1.210 and 1.012–2.153 95%CI; +71%; and dCohen = 1.814 (Figure 2G). No other significant differences in the biomarkers’ values were found.

### 3.3. Nitrite and Nitrate Post-Race Concentration

Figure 3 shows the urinary concentration of NO metabolites (NOx = Nitrites (NO_2_) + Nitrates (NO_3_)) and NO_2_ levels (μM) measured. In BCS starting from a NOx total level of 175.8 ± 130.80 μM, the concentration increased, but not significantly post-race: 281.90 ± 133.41μM; (q-value; Lower and Upper 95% Coefficient Interval (CI); delta % respect to pre-race; and size effect dCohen, respectively) q > Q; 95%CI 103.3–248.2; 208–355.8; +60%; and dCohen = 0.803 (Figure 3A). As can also be observed in the figure, no significant changes in the NO_2_ were recorded (post- vs. pre-race): 1.73 ± 10.72 vs. 2.11 ± 1.11 μM; q > Q; 1.327–2.128 and 1.501–2.730 95%CI; −18%; and dCohen = 0.415 (Figure 3B). No significant differences in the CTR group were found.

### 3.4. Creatinine, Neopterin, and Electrolytes Post-Race

In BCS, significant differences were found post- vs. pre-race in creatinine 1.48 ± 0.46 vs. 1.08 ± 0.45 g.L^−1^; q < Q; 1.232–1.74 and 0.8309–1.334 95%CI; −6%; and dCohen = 0.879 (Figure 3C). No significant differences were found in neopterin 111.40 ± 50.52 vs. 102.20 ± 46.29 μmol.mol^−1^ creatinine; q > Q; 83.47–139.40 and 76.52–127.80 95%CI; +9%; and dCohen = 0.0189 (Figure 3D). Despite the decrease, post-race, no significant differences were found for uric acid (7.45 ± 3.32 vs. 11.66 ± 5.58 mg.dL^−1^; q > Q; 5.608–9.290 and 8.571–14.76 95%CI; −36%; and dCohen = 0.919) (Figure 3E). No significant differences in the CTR group were found.

Table 2 reports the BCS group values of urea, Na, K, Cl, P, Mg, and Ca, measured pre- and post-race. Changes in the delta percent are also reported. After the race, we observed significant changes in Na (−21%), K (−29.9%), Cl (−27%), and Mg (−1%) while urea, P, and Ca did not show any difference.

### 3.5. Post-Race Appetite Hormones

There was a decrease in leptin 11.61 ± 3.28 vs. 7.85 ± 1.74 ng.mL^−1^; q > Q; 6.887–8.823 and 9.799–13.43 95%CI; −32%; and dCohen = 1.435 (Figure 4A) and a significant increase in ghrelin: 247.70 ± 124.00 vs. 377.80 ± 140.40 pg.mL^−1^; q = 0.0022; 300.10–455.60 and 179–316.3 95%CI; +53%; and dCohen = 0.982 (Figure 4B). Salivary levels were measured post- vs. pre-race in BCS. IGF-1 did not show statistical differences: 198.30 ± 91.64 vs. 177.90 ± 81.79 pg.mL^−1^; q > Q; 241.9–23.2 and 147.6–132.6 95%CI; −10%; and dCohen = 0.235) (Figure 4C).

### 3.6. Psychometric and Physical Scales in BCS

The total quality of recovery reported a value of 15.80 ± 2.4, showing good restoration in psycho-physiological recovery. The total mood score of the POMS scale was recorded before the race and −0.07 ± 9.16 was reported. The rating of perceived exertion (RPE) reported a score of 14.20 ± 1.9.

**Figure 5:**
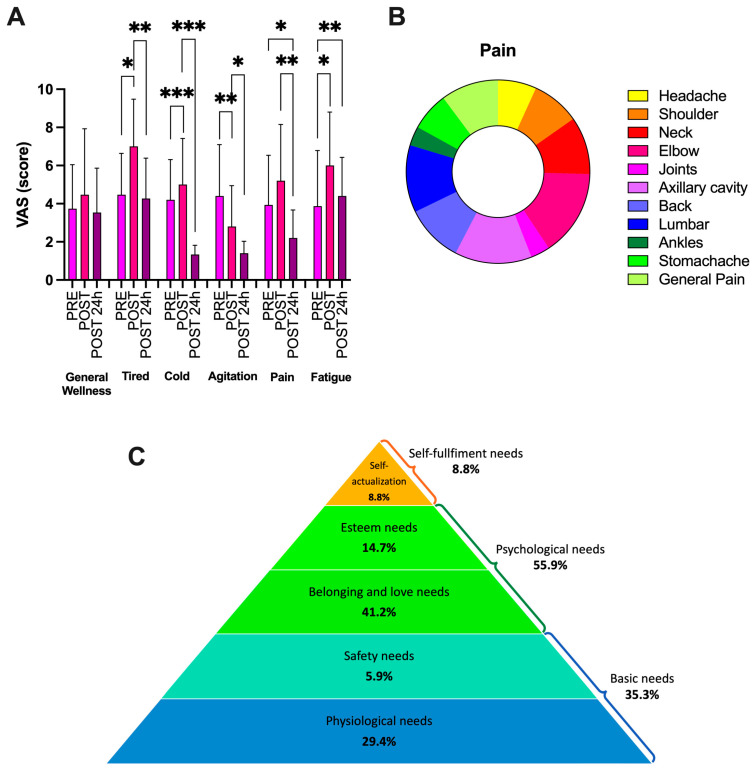
(**A**) VAS scores for general wellness, tiredness, cold, agitation, pain, and fatigue assessed pre-, post-race, and 24 h after the race. Statistically significant difference comparisons are displayed: * *p* < 0.05; ** *p* < 0.01 *** *p* < 0.001. In (**B**), the chart shows the pain area with respect to the display of part-to-whole relationships. In (**C**), Maslow’s pyramid is displayed (based on Maslow, 1954, https://www.simplypsychology.org/maslow.html; last access 11 April 2023).

Particularly, we observed percentage changes post-race vs. pre-race: general wellness: +20%; tired: +48%, cold: +15%, anxiety: −40%, pain: +32%, and fatigue: +55%. (See Figure 5A). Post-race, muscle pain was very important. Figure 5B shows the chart with the percentages of body segments affected by pain. With respect to the immediately measured post-race parameters, at 24 h post-race, significant reductions in tiredness (−38%), cold (−75%), agitation (−50%), pain (−58%), and fatigue (−27%) was found.

Maslow’s pyramid reports the results of human motivation referred to in the dragon boat activity. As shown in Figure 5C, psychological needs are very important for these women; in fact, belonging and love needs with esteem needs comprise about 56% of the responses. In addition, the calculated EORTC QLQ-C30 quality-of-life scores for global, functional, and symptom domains resulted in 80.28 ± 11.94, 82.27 ± 9.41, and 49.84 ± 10.98, respectively.

Finally, in Table 3, the perceived benefits produced by the dragon boat activity are reported.

**Figure 6:**
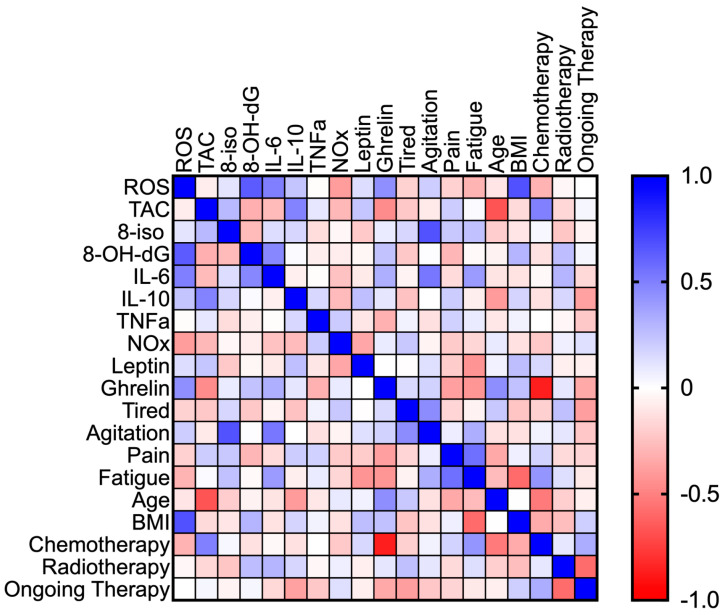
Heatmap of the correlation matrix post-race. Mean of side-by-side replicates. The bar on the right side of the map indicates the color legend (from −1 to 1) of the Spearman correlation coefficients calculated for each couple of variables in the matrix. The Spearman rank correlation test was used. *p* < 0.05 value was accepted as significant.

In the BCS group post-race, all biomarkers, VAS items, age, BMI, past chemotherapy, radiotherapy, and actual therapy were correlated against each other to statistically evaluate potential overlaps of compartment markers. High correlation coefficients were found in: ROS production vs. 8-OH-dG (*p* = 0.121); TAC vs. age (*p* = 0.008); 8-iso-PGF2-a vs. agitation item (*p* = 0.008); IL-6 vs. the agitation item (*p* = 0.043); Ghrelin vs. chemotherapy (*p* = 0.0003); score pain vs. fatigue item (*p* = 0.032); BMI vs. ROS production (*p* = 0.007); and BMI vs. the fatigue item (*p* = 0.002) (Figure 6).

## 4. Discussion

Fatigue, lymphedema, and chronic pain are some of the common symptoms referred to by breast cancer patients both in the short- and long-term, affecting quality of life. They can be counteracted, in part, by the numerous benefits that physical exercise has on health, associated with the reduction of OxS that can be a direct or indirect cause, and which, in any case, contributes to exacerbating the aforementioned side effects [7]. Based on the results, Joaquim et al. [37] drew the conclusion that physical exercise is an effective strategy that positively affects breast cancer survivors’ quality of life, cardiorespiratory fitness, and body composition

A review article [38] included a variety of studies, such as randomized controlled trials, pilot studies, and clinical trials. The studies were reviewed for three major outcomes: changes in breast cancer mortality, physiological functions, and metabolic biomarkers. Many of the studies suggest that BCS benefit from engaging in physical activity, but some studies are limited in their ability to provide adequate evidence due to relatively small sample sizes, short intervention periods, or high attrition. Based on epidemiological evidence, recent studies have demonstrated that those BCS who engage in physical activity significantly lowered their risk of breast cancer mortality and improved their physiological and immune functions. Some studies demonstrate changes in metabolic biomarkers, such as insulin and insulin-like growth factors. However, further investigation is required to support these findings because these results are not consistent. As concerns the relationship between cytokines and exercise, in a study concerning the effects of an 8-week (aerobic+strength) exercise training program, no major changes in the basal cytokine levels of BCS were induced [39].

A previous study concerning endurance training activity in breast cancer patients addresses muscular strength and quality of life, rather than metabolic and oxidative stress responses [40]. By contrast, few studies were found on BCS and dragon boat paddling [18] and none particularly regarding a single endurance exercise.

Endurance exercise increases the oxygen consumption rate, which, in turn, increases ROS production [41,42], so antioxidant defenses are mostly required to protect cells from oxidative damage as overproduction of ROS potentially damages cells, particularly macromolecules as we can see in the rise of lipid peroxidation and DNA damage [43]. Therefore, from the analysis of the literature arises the question of whether endurance exercise is safe [44].

In cancer cells, the excessive ROS lead to an increase in the antioxidant capacity, contrasting with the apoptotic function of oxidative stress [45]. ROS are mainly produced in mitochondria as by-products of the electron transport chain (ETC) and for this reason, the cell also produces antioxidant scavengers to avoid oxidative stress and the consequent damage due to excessive ROS amounts [46].

In the present study, 15 healthy females (HFS group, comparable for age, BMI, and fitness level) were assumed as a control group: just the resting data showed up a significant increase of the biomarker levels in the studied BCS group, as previously reported [18]. The aim of this study was to evaluate with a semi-invasive technique the oxy-inflammation biomarkers before and after an endurance non-competitive race of about 30 km in a group of women with a previous breast cancer and, therefore, with an increased initial oxy-inflammatory status. Our results show a significant increase in ROS production after the race, as well as biomarkers of lipid peroxidation and DNA damage, while the total antioxidant capacity decreased. Also, the inflammatory markers increased. Therefore, this study confirms that endurance and very prolonged (>5 h) exercise caused an increase in ROS production, oxidative stress, and inflammatory status.

In our study, IL-6, TNF-α, and IL-10 recorded at the baseline (pre-race) are in agreement with the values reported in the literature [47], and they significantly increased post-race. This can be explained by the fact that we detected these cytokines after a 5 h race of about 30 km. Vigorous and demanding exercise triggers a strong inflammatory reaction, marked by a rise in the overall count of circulating neutrophils, monocytes, and oxidative stress levels. This, in turn, promotes cytokine production from various cell types and escalates the inflammatory cascade [46]. IL-10 is an anti-inflammatory cytokine that has a strong downregulatory effect on the secretion of proinflammatory cytokines such as IL-1, IL-1*β*, and TNF-*α*. The magnitude of the increase in the concentration of IL-10 seems to be mainly dependent on the release of IL-6 and the exercise duration [8]. During exercise, working skeletal muscles release anti-inflammatory cytokines, like IL-6, which significantly reduce symptoms such as fatigue and poor quality of life.

Schneider and colleagues (2012) [48] reported that, following treatments, in breast cancer survivors, physical capacity often declines, and the inflammatory response to exercise may be more pronounced due to the cumulative effects of cancer therapy and/or immune and/or antioxidant systems. According to the authors, our data showed an inflammatory state in response to the exercise, compared to the CTR group.

Another marker of pro-inflammation is urinary neopterin, mainly produced by activated macrophages and excreted by glomerular filtration. For this reason, it is also a useful marker of renal functionality [49]. No significant differences in neopterin were recorded, suggesting a preservation of renal function.

As is well known, intense exercise contributes to a rise in creatinine. However, the rise of post-race urinary creatinine values, remained always within the normal range, which leads us to deduce an increase due to physical exercise, when considering the endurance race, and not due to a renal disease.

Skeletal muscle continually produces ROS and NO derivatives that increase dramatically in muscle tissue during exercise. In fact, muscle contractions are predicted to stimulate ROS production in active fibers, making skeletal muscle a primary source of ROS during exercise. Such ROS generation is linked to oxidative damage, accelerating muscle fatigue, as well as the activation of signalling pathways that drive exercise-induced adaptations [50]. As reported in a study by Ting-Ting Lee and colleagues [51], acute exercise using a canoe/kayak induces oxidative stress with alterations in plasma concentrations of superoxide dismutase, catalase, and TBARS in athletes, and this can have short-term effects on the immune function. Despite, as reported by other authors, during extreme physical-sporting activities, such as endurance exercise, the generated ROS amount being able to exceed the body’s antioxidant capacities [52,53,54,55], such an ROS increase can, at the same time, positively influence the antioxidant compartments and may be considered a direct reaction to a physical activity-induced oxidative environment [18].

We also observed an increase in total NO metabolites, even if it resulted as not being significant. NO produced during exercise induced vasodilation, accompanied by an increase of blood flow; however, when excess NO is produced through the up-regulation of inducible NOS (iNOS), toxic peroxynitrite is produced through a reaction with superoxide [56].

Physical activity raises energy expenditure, promoting amino acid catabolism, especially that involved in energy need [57], leading to an increase of urea excretion [58]. Despite that, we did not observe significant changes in urea. Significant reductions were recorded in urinary Na and K after the race. Urinary Na reduction, also observed by some authors [59,60], may be the consequence of a reduction in glomerular filtration rate and/or an increase of Na reabsorption. This increased tubular reabsorption seems to play a key role in urinary Na reduction after exercise [61]. K decrease may be related to a reduced Na level by increasing the sodium–potassium exchange [62] and sustaining physical effort [63]. Urinary Na reduction is followed by urinary Cl reduction depending on the on-exercise intensity [64,65]. Mg reduction may be related to energy need to sustain the physical effort. Indeed Mg, in its ionic form Mg^2+^, reacts with adenosine triphosphate (ATP) to obtain the Mg-ATP complex [66]. Furthermore, the Mg decrease may be related to the inflammatory status [67] and oxidative stress onset [68]. Finally, we did not observe significant differences in uric acid values, one of the most abundant circulating antioxidants.

After the race, we observed a decrease in leptin and a significant increase in ghrelin, indicating an increase in appetite due to a reduction of energetic reserves. A recent study [69] has investigated the effects on appetite of resistance exercise in breast cancer survivors. They concluded that a single bout of resistance exercise did not influence changes in appetite and that there is no difference between BCS and healthy subjects. The results from the current study agree with previous research that demonstrates decreases in leptin concentrations with exercise and changes are dependent on duration, intensity, and exercise mode [70]. The previous literature has reported that exercise bouts shorter than 60 min may not alter leptin concentration [71]. Thereafter, the difference observed may be due to the long duration (5 h) and intensity (30 km paddling/rowing race) of the exercise performed.

As anticipated in the introduction, numerous studies underscore the dragon boat’s ability to improve health-related quality of life (HRQoL) [72]. Research has shown that participants often report increased self-esteem, body image satisfaction, and reduced anxiety and depression after participating in dragon boat activities [73]. Our data are in agreement with this; in fact, a significant improvement was reported post-race on the VAS scale for the items of general wellness: +20% and relaxation: +40%, even though the dragon race still caused an increase in physical pain: +32% and physical fatigue: +55%. The sense of accomplishment achieved through racing contributes to greater emotional resilience and psychological well-being [74]. As a group activity, dragon boat racing fosters strong social bonds, which are known to buffer stress and contribute to emotional health [73].

As we can see for the questionnaire on perceived benefits from the dragon boat activity, the women involved in this study were strongly motivated to practice this kind of sport, not only for physical benefits, but also for psychological reasons. On the other scale submitted, we can also observe a good restoration of psycho-physiological recovery and the importance of the psychological needs that emerge in Maslow’s pyramid. Finally, the QoL scores recorded by EORTC provided essential insights into the relative well-being of our tested BCS participants. BCS had a good quality of life, with symptoms attributable to pathology, but which did not interfere with their daily activities. Indeed, they were determined to carry out group activities; in fact, the functional scales showed an excellent score. These benefits are also confirmed by several studies on the dragon boat [75]. Finally, through the analysis of several studies on the dragon boat, it has emerged that the prescription of physical exercise implicit in this discipline is particularly suitable for breast cancer survivors. This is due to the physical benefits deriving from the type of training that contributes not only to reducing side effects, but also to its ability to actively involve patients, creating a strong team spirit which plays an important role in improving psychological well-being [76].

### Limitations

One limitation of this study was the inability to include more crews participating in the race. Due to logistical challenges, such as the geographical dispersion of participants across different cities, it was not feasible to gather data from a broader range of crews. Moreover, another limit might be linked to the intrasubject variability (i.e., age, previously undergoing therapy, years from diagnosis). This restriction limited the generalization of findings, as a larger and more diverse sample could provide a more robust representation of the population.

No sweat collection was conducted due to the difficulty of the technical procedure as indicated by Shirreffs and Maughan [77]. Furthermore, Constantinescu and Hilman [78] reported that the sweat test’s reliability may be in jeopardy, owing to several factors, including a lack of uniformity in the preanalytical pilocarpine iontophoresis stimulation and subsequent sweat collection. Despite these limitations, valuable data on the biological responses of oxidative stress, inflammation, and hormonal changes in breast cancer survivors who participated in an endurance dragon boat race were obtained. The study provides new insight into the role played by these physical challenges in affecting the investigated biomarkers on this specific population. All these findings are crucial for understanding the physiological impact of intense exercise on cancer survivors, potentially providing future recommendations for physical activity and recovery strategies in this group.

Finally, it goes without saying that data on post-race recovery and long-term follow up are suggested to advance the study.

All these limitations made this research an observational, non-invasive, pilot study.

## 5. Conclusions

In conclusion, the increases in inflammatory biomarkers and oxidative stress suggest a need for careful monitoring or adjustments to the intensity and duration of physical activity, particularly in BCS. Indeed, these findings should be viewed with caution, since exercise can benefit rehabilitation, but excessive physiological stress may be harmful for these kind of patients.

Despite the significant limitations in the study design and data analysis that made it pilot research, the results reported here encourage us to undergo further analysis conducted with an increase in the number of BCS, as well as with obtaining more data from healthy subjects collected when performing the same exercise. However, our findings highlighted, all the same, that dragon boat racing offers to BCS a holistic approach to post-treatment recovery, addressing critical emotional and social needs. At the same time, the evidence that dragon boat paddling actively involves patients, creating a strong team spirit which plays an important role in improving their psychological well-being, strongly supports its integration into rehabilitation programs, providing survivors with an empowering pathway to rebuild their lives and improve their quality of life. Finally, we want to highlight that our study is an observational, non-invasive, pilot investigation and does not intend to provide a definitive recommendation regarding physical activity in breast cancer survivors. On the contrary, we emphasize the caution required when prescribing exercise to these patients.

## Figures and Tables

**Table 1:** BCS group characteristics. IDC = Invasive Ductal Carcinoma of the breast; DCIS = Ductal Carcinoma in Situ.

Subjects n = 15	Mean ± SD
**Age (years)**	55.73 ± 4.96
**Body weight (kg)**	65.27 ± 12.60
**Height (cm)**	163.3 ± 4.2
**BMI (kg.m^−2^)**	24.43 ± 4.30
**Diagnosis age (years)**	46.9 ± 5.6
**Histopathological Characterization:**	
IDC	86% (n = 13)
DCIS	13% (n = 2)
**Years after surgery**	8.8 ± 4.9
**Axillary lymph node dissection**	46.7% (n = 7)
**Chemotherapy**	53.3% (n = 8)
**Targeted drugs**	26.7% (n = 4)
**Hormone therapy**	73.3% (n = 11)
**Radiotherapy**	66.7% (n = 10)
**Ongoing therapy**	33.3% (n = 5) hormone therapy (Aromatase inhibitors, 2.5 mg/die) 6.7% (n = 1) other therapies (Levothyroxine for Hypothyroidism)
**Metastases**	0%
**Recurrences**	6.7% (n = 1)
**Experience on dragon boat (years)**	Range 2–5

**Table 2:** Levels of urea, Na, K, Cl, P, Mg, and Ca measured pre- and post-race in BCS group. Changes in delta percent are reported too.

Parameter	Pre-Race	Post-Race	*p* Value	Δ%
**Urea (mg/dL)**	721.3 ± 291.3	823.0 ± 480.7	n.s.	+14
**Na (mmol/L)**	78.5 ± 35.8	62.0 ± 41.3	*	−21
**K (mmol/L)**	67.3 ± 36.5	47.2 ± 34.2	*	−30
**Cl (mmol/L)**	87.3 ± 30.8	64.1 ± 37.8	**	−27
**P (mg/dL)**	64.1 ± 32.6	50.9 ± 43.5	ns	−21
**Mg (mg/dL)**	8.4 ± 5.2	8.3 ± 8.2	*	−1
**Ca (mg/dL)**	13.1 ± 9.3	10.9 ± 6.7	ns	−17

Results are expressed as mean ± SD; n.s. not significative; * *p* < 0.05, ** *p* < 0.01; differences compared to pre-race.

**Table 3:** Perceived benefits of dragon boat activity.

Life Enhancement Sub-Scale	
My disposition is improved by exercise	3.6 ± 0.5
Exercise helps me sleep better at night	2.3 ± 0.7
Exercise helps me decrease fatigue	3.1 ± 0.9
Exercising improves my self-concept	3.1 ± 1.0
Exercising increases my mental alertness	2.5 ± 0.5
Exercise allows me to carry out my normal activities without becoming tired	3.0 ± 0.9
Exercise improves the quality of my work	3.0 ± 0.9
Exercise improves overall body functioning for me	3.0 ± 0.9
**Physical performance sub-scale**	
Exercise increases my muscle strength	3.6 ± 0.6
Exercise increases my level of physical fitness	3.1 ± 0.5
Muscle tone is improved by exercise	3.3 ± 0.6
Exercising improves functioning of my cardiovascular system	2.7 ± 0.5
Exercise increases my stamina	3.2 ± 0.5
Exercise improves my flexibility	2.9 ± 0.5
My physical endurance is improved by exercising	3.1 ± 0.5
Exercise improves the way my body looks	2.3 ± 1.0

## Data Availability

Data are available at request from the authors.

## Abbreviations

BCS	Breast Cancer Survivors
BMI	Body Mass Index
DBP	Diastolic Blood Pressure
EORTC	European Organization for Research and Treatment of Cancer
EPR	Electron Paramagnetic Resonance
HPLC	High-pressure Liquid Chromatography
HR	Heart Rate
IL-6	Interleukin-6
NO	Nitric Oxide
NOx	Nitric Oxide metabolites
OxS	Oxidative Stress
POMS	Profile of Mood States
QLQ-C30	Quality of Life Group Core-30
QoL	Quality of Life
SBP	Systolic Blood Pressure
ROS	Reactive Oxygen Species
RPE	Perceived Exertion scale
TAC	Total Antioxidant Capacity
TQR	Total Quality of Recovery
TNFα	Tumor Necrosis Factor-alfa
VAS	Visual Analog Scale
8-iso-PGF2α	8-isoprostane-PGF2alfa
8-OH-dG	8-Hydroxy-2′-deoxyguanosine
